# Primary Idiopathic Frosted Branch Angiitis

**DOI:** 10.18502/jovr.v15i3.7462

**Published:** 2020-07-29

**Authors:** Shahin Jahani Maleki, Maryam Dourandish, Seyedeh Maryam Hosseini

**Affiliations:** ^1^Eye Research Center, Mashhad University of Medical Sciences, Mashhad, Iran; ^2^Retina Research Center, Mashhad University of Medical Sciences, Mashhad, Iran

##  Presentation

A five-year-old boy presented to the ophthalmology emergency department with a three-day history of sudden onset of severe visual impairment in both eyes. He experienced an upper respiratory tract infection (URTI) two weeks before the presenting complaint. On initial ophthalmic examination, his visual acuity was hand motion with projection OU. He had no relative afferent pupillary defect, and had normal intraocular pressure. The visual reduction was not associated with pain or injection. Slit-lamp examination of both eyes showed mild anterior chamber (AC) reaction (1–2+ cells), moderate bilateral presence of vitreous cells (2–3+ cells), but no keratic precipitates.

**Figure 1:**
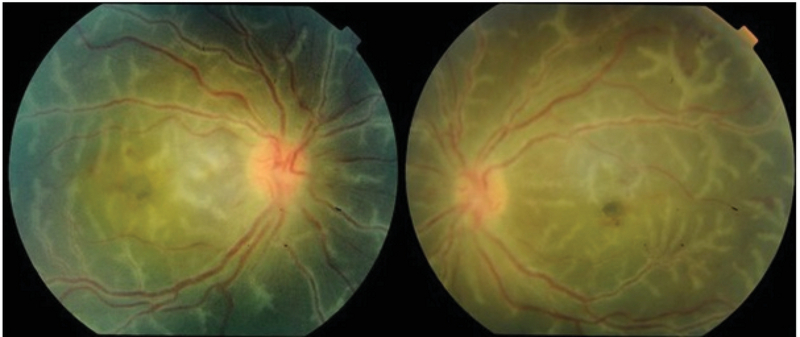
Bilateral primary idiopathic frosted branch angiitis in a 5-year-old boy. Note the prominent, florid, translucent, retinal perivascular sheathing affecting both the venules and arterioles. The sheathing originates from the posterior pole and extends up to the periphery.

A dilated fundus examination of both eyes revealed mild vitreous haziness and bilateral symmetrical and widespread retinal vasculitis. There was a prominent and florid translucent retinal perivascular infiltration that predominantly affected the venules, starting from the posterior pole and extending up to the periphery. Bilaterally, mild to moderate papillitis and severe macular edema was noted without any obvious retinal hemorrhages (Figure 1).

**Figure 2:**
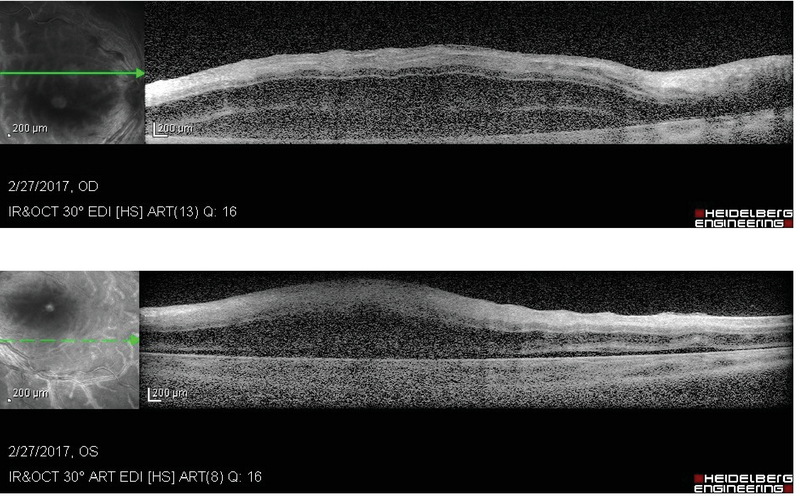
Spectral domain optical coherence tomography (SD- OCT) of the posterior pole reveals highly reflective retinal layers suggestive of diffuse edema and multifocal exudative retinal detachments.

Spectral-domain optical coherence tomography (SD-OCT) of both eyes showed high reflectivity in the inner retina suggestive of intracellular edema, and multifocal neurosensory detachments were seen at the posterior pole (Figure 2).

The patient was admitted for an extensive work-up. Anterior chamber paracentesis was performed to detect the possible presence of viral pathogens by polymerase chain reaction (PCR). Consultations with the pediatrics, rheumatology, infectious diseases, and neurology specialists were requested. Laboratory tests were negative for infectious, rheumatological, and malignant disorders.

Results of real-time PCR of the aqueous humor sample was negative for cytomegalovirus, herpes simplex virus, varicella zoster, and TB. Brain MRI examination revealed no pathologies such as multiple sclerosis and infiltrative diseases.

**Figure 3:**
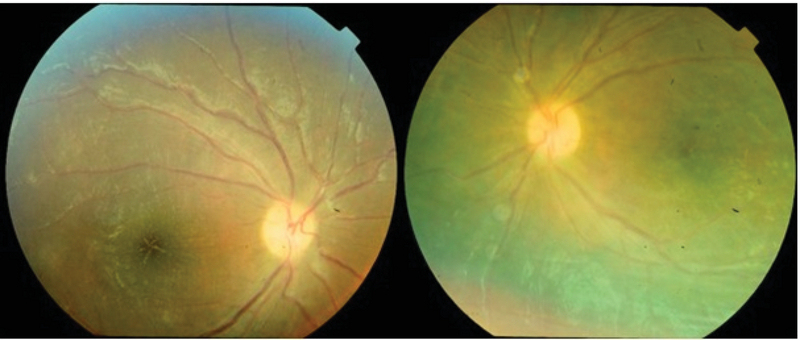
At the end of the 2${}^{\mathrm{nd}}$ week of treatment, complete resolution of the vascular sheathing and exudates is observed. Note the pigmentary macular change in both eyes.

**Figure 4:**
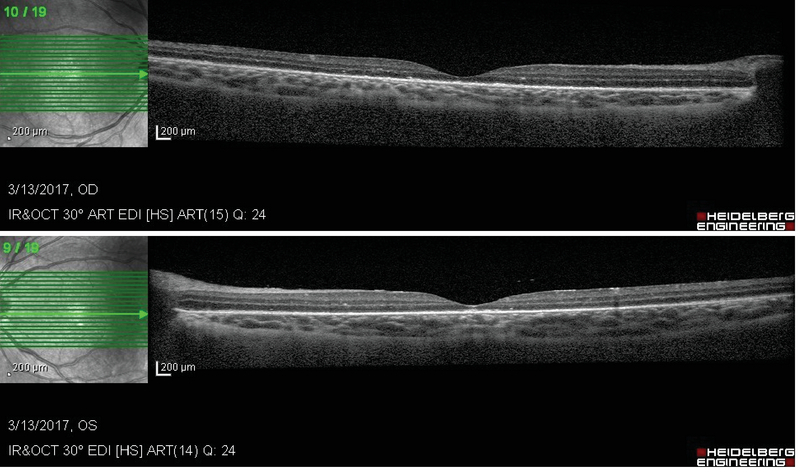
Macular OCT image shows complete resolution of the retinal exudates and subretinal fluids. The central macular thickness of the right and left eyes is 188 and 183 µm, respectively.

The severe vision-threatening condition was diagnosed to be primary frosted branch angiitis, and empirical treatment with oral prednisolone (1 mg/kg/day) and topical corticosteroids was initiated. On the fourth day of the treatment, a significant therapeutic response was observed. At the end of the second week, vascular sheathing and exudates resolved completely (Figure 3) and visual acuity in both eyes improved to 3/10 (+0.5 LogMAR). SD-OCT image showed complete resolution of the subretinal fluid (Figure 4).

**Figure 5:**
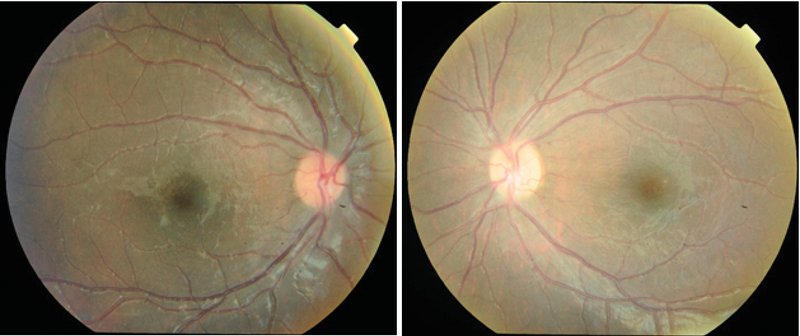
Fundus photography after 12 months of treatment shows mild granular macular pigmentation.

At the three-month follow-up, visual acuity improved to 7/10 (+0.15 LogMAR) in both eyes; no active inflammation or vasculitis was observed. Ophthalmic examinations at the 6 and 12-month follow-ups showed bilateral improvement of vision to 9/10 (+0.04 LogMAR), without any recurrence or significant sequelae. The only abnormal finding was mild pigmentary macular change (Figure 5).

##  Discussion

Frosted branch angiitis can be idiopathic or secondary to ocular or systemic conditions. Because there are various secondary causes, it is important to perform an extensive work-up to exclude other causes of vasculitis before making a diagnosis of primary idiopathic retinal vasculitis. In the present case, possible underlying conditions such as infectious, rheumatological, or malignant causes were ruled out after extensive evaluation.^[1,2,3,4]^

This report re-emphasizes the considerable response of this rare disease to corticosteroid therapy without any significant visual consequences.

##  Financial Support and Sponsorship

Nil

##  Conflicts of Interest

There are no conflicts of interest.
